# High risk of misinterpreting liver and spleen stiffness using 2D shear-wave and transient elastography after a moderate or high calorie meal

**DOI:** 10.1371/journal.pone.0173992

**Published:** 2017-04-04

**Authors:** Maria Kjærgaard, Maja Thiele, Christian Jansen, Bjørn Stæhr Madsen, Jan Görtzen, Christian Strassburg, Jonel Trebicka, Aleksander Krag

**Affiliations:** 1 Department and Gastroenterology and Hepatology, Odense University Hospital, Odense, Denmark; 2 Odense Patient data Explorative Network (OPEN), Odense, Denmark; 3 Department of Clinical Research, University of Southern Denmark, Odense, Denmark; 4 Department of Internal Medicine I, Universitätsklinikum Bonn, Bonn, Germany; 5 European Foundation for the Study of Chronic Liver Failure (EF Clif), Barcelona, Spain; University of Navarra School of Medicine and Center for Applied Medical Research (CIMA), SPAIN

## Abstract

Food intake increases liver stiffness, but it is believed that liver stiffness returns to baseline two hours after a meal. The aim of this study was to investigate the impact of different sized meals on liver stiffness. Liver and spleen stiffness was measured with transient elastography (TE) and real-time 2-dimensional shear wave elastography (2D-SWE). Patients ingested a 625 kcal and a 1250 kcal liquid meal on two consecutive days. We measured liver and spleen elasticity, Controlled attenuation parameter (CAP) and portal flow at baseline and after 20, 40, 60, 120 and 180 minutes. Sixty patients participated, 83% with alcoholic liver disease. Twenty-eight patients had METAVIR fibrosis score F0-3 and 32 patients had cirrhosis. Liver stiffness, spleen stiffness and CAP increased after both meals for all stages of fibrosis. False positive 2D-SWE liver stiffness measurements caused 36% and 52% of patients with F0-3 fibrosis to be misclassified with higher stages of fibrosis after the moderate and high caloric meal. Likewise, 10% and 13% of compensated cirrhosis patients were misclassified with clinically significant portal hypertension after the two meals. We observed similar misclassification rates with TE. After three hours, liver stiffness remained elevated more than 20% from baseline in up to 50% of patients. In conclusion: Liver stiffness, spleen stiffness and CAP increase after a meal across all stages of fibrosis and across elastography techniques. Up to half of patients may be misclassified with higher stages of fibrosis, if they are assessed after less than three hours fasting period.

## Introduction

Cirrhosis is the 8^th^ leading cause of life years lost in the United States and are responsible for 1.2 million deaths every year world-wide.[1] Treatment of underlying cause and co-factors might prevent progression and complications; accurate and timely diagnosis of chronic liver disease is therefore of crucial importance. For the diagnosis and staging of chronic liver disease information on the amount of fibrosis, portal hypertension and steatosis, especially in case of alcoholic and non-alcoholic fatty liver disease, are important.

The reference non-invasive method to stage liver fibrosis is transient elastography (TE).[2–4] TE was introduced in Europe in 2003 and FDA approved in 2013 and is now available in more than 70 countries. TE is recommended in clinical guidelines to support decisions regarding risk of liver disease, need of follow-up and indication for treatments.[5–7]. Thus reliable measurements are essential.[8]

Spleen stiffness is proposed as a marker of clinically significant portal hypertension and esophageal varices [9, 10] and controlled attenuation parameter (CAP) is a novel non-invasive marker of liver steatosis.[11] All these techniques are based on the physical properties of the liver and spleen, which might be confounded by factors other than fibrosis or steatosis. Overestimation of liver stiffness may affect clinical decision-making and lead to futile invasive interventions such as upper endoscopy and liver biopsy.

Food intake has been shown to increase liver stiffness.[12–17] However, several points are unexplored; such as the role of the size and caloric content of the meal on liver stiffness, the influence of a meal in patients without severe fibrosis or cirrhosis and the time needed for liver stiffness to normalize, whether the effect of a meal is the same across different elastography techniques, whether the effect of a meal is the same in other etiologies than chronic viral hepatitis, and whether a meal also affects spleen stiffness and CAP values.

These limitations should be resolved before clear, general recommendations can be made to avoid false positive liver stiffness measurements.[2]

The aim of this study was to evaluate the effect of a moderate caloric and a high caloric meal on the risk of misclassification of fibrosis, portal hypertension and steatosis in patients with chronic liver disease using 2D-SWE, TE and CAP.

## Patients and methods

### Patients

We recruited patients consecutively from the outpatient liver clinics at Odense University Hospital in Denmark and Bonn University Hospital in Germany.

We included patients between 18 and 80 years with: (I) liver biopsy within 18 months of inclusion, except in patients with a diagnosis of cirrhosis based on characteristic biochemical, ultrasonographic and/or endoscopic findings, (II) prior or current excess alcohol use for more than one year or anti-HCV positivity for more than six months, and (III) ability to read and write Danish/German. We excluded patients with: (I) cholestasis or right heart failure evidenced by ultrasound, (II) hepatic inflammation defined as transaminase level more than three times the upper level, (III) prior failure to acquire TE or SWE measurements, (IV) primary or secondary hepatic malignancy, (V) severe comorbidity with an estimated survival < 12 months and (VI) liver transplantation.

Included patients were divided into two groups according to their METAVIR fibrosis score: fibrosis (METAVIR F0-3), and cirrhosis (METAVIR F4).

The regional ethics committee of Southern Denmark (project-ID: S-20130087) and ethical Committee of University of Bonn (121/14) approved the study protocol. The study honored the World Medical Association Declaration of Helsinki’s ethical principles for medical research involving human subjects. All patients consented in writing before study inclusion.

### Study protocol

**Fig 1:**
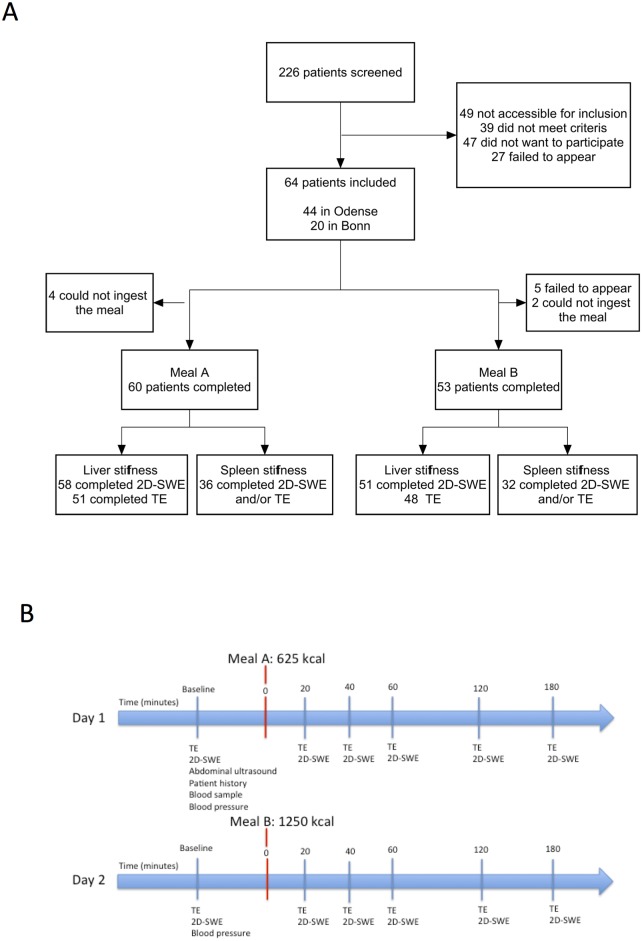
Study flow (A) and study design (B).

The patients were investigated on two separate days days within the same week, after at least a three-hour fasting period. The study design is outlined in Fig 1.

On the first day, after baseline measurements, the patients ingested a 250 ml liquid meal containing 625 kcal and consisting of 49% carbohydrates, 36% fat and 15% protein (Nestlé Ressource 2.5 Compact) (Meal A). On day two, patients ingested another liquid meal of twice the caloric and volumetric size (500 ml and 1,250 kcal) (Meal B). Both meals were ingested within five minutes.

Baseline and post-meal measurements included liver stiffness and spleen stiffness with 2D-SWE and TE, CAP, portal vein diameter and portal venous blood flow at 20, 40, 60, 120 and 180 minutes after meal ingestion. We calculated portal venous flow by this formula: Flow (ml/min) = (portal vein diameter (cm)/2)^2^ x π x 60 x portal velocity (cm/s).

We characterized patients with gender, age, body mass index, METAVIR fibrosis score, abdominal ultrasound and liver blood tests.

### Liver and spleen elastography

In both participating hospitals an experienced operator with >500 TE exams performed all liver stiffness and spleen stiffness measurements with the equipment Aixplorer (Supersonic Imagine, France) for 2D-SWE and FibroScan 502 touch (Echosens, France) for TE and CAP.

2D-SWE was performed according to previous descriptions,[18] with a curved abdominal probe (3–5 Hz). We performed the measurements in a right (liver) and left (spleen) intercostal space, during expiratory breath-hold, aiming for a stable, homogeneous elastogram with complete filling both spatially and temporally. We considered liver stiffness by 2D-SWE a success if at least three measurements were acquired using a Q-box≥15mm and depth < 5.6 cm.[18, 19] Spleen stiffness was considered a success if at least one measurement was acquired using a Q-box ≥10mm and with a SD<30% of the mean. 2D-SWE exams were reported as the mean of the total number of measurements.

TE was performed according to standard,[20] in a right (liver stiffness) or left (spleen stiffness) intercostal space, with the patient in the supine position, hands above his/her head, during expiratory breath-hold. TE results for liver stiffness were considered reliable if 10 valid measurements were acquired with an interquartile range below 30% of the median liver stiffness, if the median exceeded 7.1.[21] Spleen stiffness with TE were measured with the FibroScan M probe under ultrasound guidance and was considered a success if 10 valid measurements were acquired.[22] In each patient, the probe used for baseline measurements on Day 1 (XL or M), was used for all subsequent measurements on Day 1 and 2.

### Statistical analysis

Summary statistics are reported as means and standard deviations or medians and interquartile ranges, depending on distribution. For group comparisons, we used a t-test for normal distributed data and the Wilcoxon rank sum test for non-normal distributed data. Results with a p-value below 0.05 were considered statistically significant.

For the analysis of how a meal ingestion affected the liver stiffness, we labeled patients as misclassified if their liver stiffness crossed a predefined threshold from baseline to peak post-meal liver stiffness. We chose the thresholds according to known cut-off values for 2D-SWE and TE to diagnose significant fibrosis, severe fibrosis, cirrhosis and clinically significant portal hypertension. Accordingly, biopsy-proven F0-1 fibrosis was labeled as misclassified with 2D-SWE and TE if liver stiffness increased from a baseline value less than 7.1 kPa, to a post-peak value of 7.1 kPa or above.[4, 23] Fibrosis stage F2-3 were labeled as misclassified if 2D-SWE increased across a 9.2 kPa cut-off for severe fibrosis or the 13.5 kPa cut-off for cirrhosis.[23] Similarly, we used TE cut-offs of 9.5 kPa and 12.5 kPa for severe fibrosis and cirrhosis.[4] For patients with cirrhosis, we used cut-offs for clinically significant portal hypertension of 16.0 kPa assessed with 2D-SWE[19, 24] and 20 kPa assessed with TE.[5] Likewise, we labeled CAP measurements as steatosis misclassification if the CAP value crossed cut-offs of 250 dB/m and 300 dB/m.[11] In addition to labeling misclassifications, we registered whether post-meal measurements increased more than 20% from baseline.

Predictors of misclassifications and changes in liver stiffness over time were investigated in logistic regression models with patient id and meal size (Meal A vs. Meal B) as the grouping variables. For liver stiffness and CAP, we tested whether fibrosis stage and meal size predicted misclassifications and a post-meal increases of ≥20% from baseline uni- and multivariate models. All analyses were performed with the statistical software STATA version 14 (Statacorp, TX, USA).

## Results

### Patient characteristics

During a 12-month period from July 2014 to July 2015, we included 60 patients (Fig 1). Forty-two (70%) of the patients were male, the mean age was 59 years (range 39–78) and in the majority of patients (83%) the etiology of liver disease was alcohol overuse (Table 1). Twenty-eight (47%) patients belonged to the fibrosis group and 32 (53%) to the cirrhosis group.

**Table 1:** Patient characteristics.

	All (n = 60)	F0-3 (n = 28)	F4 (n = 32)	*Group difference (p)*
**Male gender**	42 (70%)	22 (79%)	20 (63%)	0.179
**Age**	58.8 (±8,9)	57.9 (±8.3)	59.5 (±9.8)	0.398
**Etiology** Alcoholic liver disease Chronic viral hepatitis C Non-alcoholic fatty liver disease Other	50 (83%) 4 (7%) 2 (3%) 4 (7%)	25 - - 3	25 4 2 1	
**Ascites**	5 (8%)	-	5 (16%)	-
**Spleen diameter (cm)**	12.3 (±2.6)	11.0 (±1.4)	13.5 (±2.9)	<0.001
**Portal vein diameter (mm)**	10.6 ± 2.1	10.9 ± 1.9	10.6 ± 3.6	0.794
**Portal venous flow (ml/min)**	919 ± 408	898 ± 356	943 ± 469	0.886
**Body Mass Index (kg/m**^**2**^**)**	27 ±8.4	27 ± 4.9	29.3 ± 10.6	0.434
**Heart rate (beat/minute)**	74.0 (±14.2)	71.3 (±14.1)	77.2 (±14.0)	0.959
**Mean Arterial Blood Pressure (mmHg)**	96.7 (±11.2)	97.9 (±12.4)	95.2 (± 9.6)	0.864
**Alanine transaminase (U/l)**	30 ± 17.5	30 ± 11	35 ± 19	0.475
**Bilirubin (μmol/l)**	12 ± 15	9 ± 3	17 ± 21	<0.001
**Gamma glutamyl transferase (U/l)**	93 ± 134.5	76.5 ± 67	136.5 ± 175	0.009
**INR**	1 ± 0.2	1 ± 0.1	1.2 ± 0.3	<0.001
**Platelet count (x 10**^**9**^**/l)**	197.4 (±90.8)	250.3 (±69.7)	148.7 (±80.8)	<0.001
**Albumine (g/l)**	41 ± 6	43 ± 2	38 ± 6	<0.001
**Alkaline Phosphatase (U/l)**	99 ± 59.6	78 ± 35	116 ± 47	<0.001

Normal distributed data are stated as mean ±SD and non-parametric data as median ± IQR. The p-values denote the significance test for between-group difference using Wilcoxons rank sum test.

INR, international normalized ratio; IQR, interquartile range; SD, standard deviation

### Changes in liver stiffness after a moderate or high calorie meal

**Fig 2:**
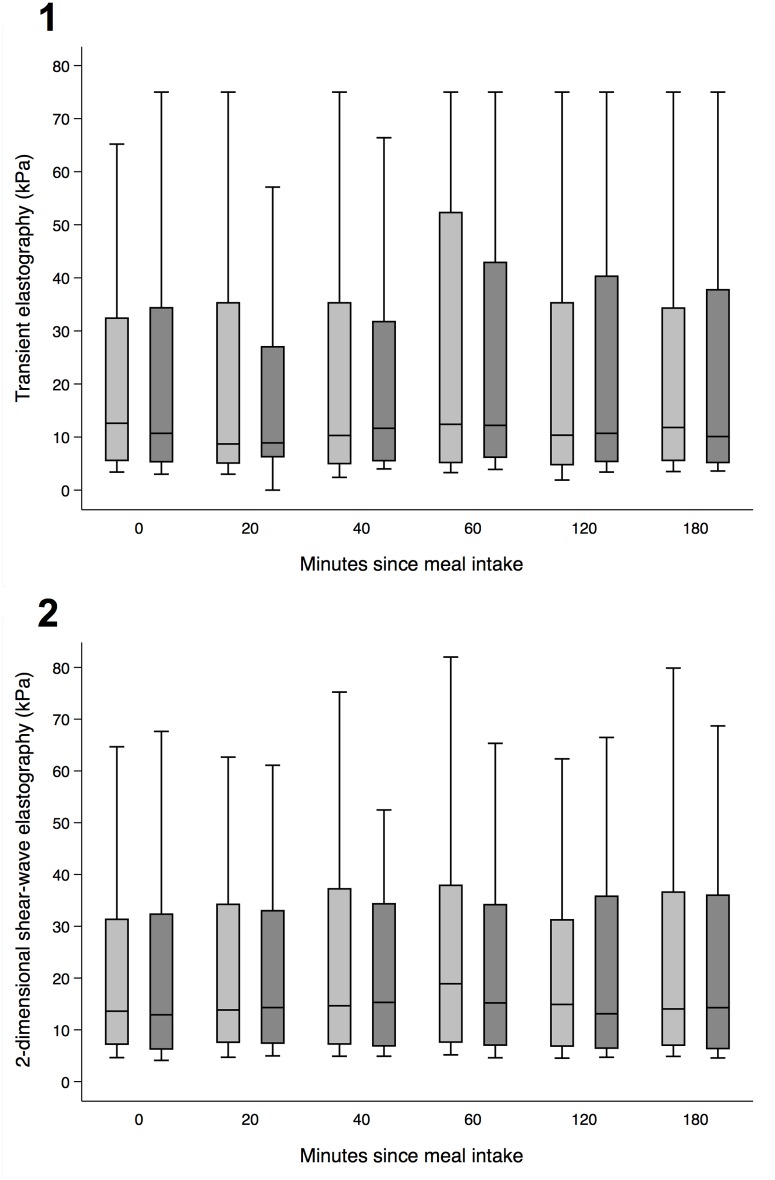
Liver stiffness at different time points. Liver stiffness after meal A (light grey) and meal B (dark grey); mean and SD of liver stiffness measured by TE (1) and 2D-SWE (2).

**Fig 3:**
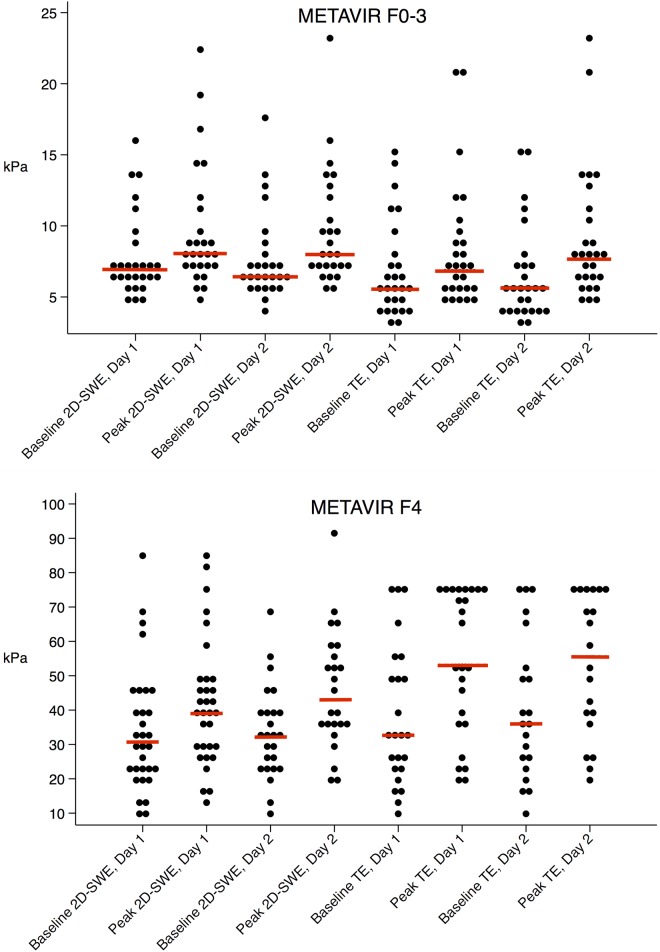
Changes in liver stiffness values. Changes from baseline to peak liver stiffness after the moderate meal (Meal A) on day 1 and high caloric meal (Meal B) on day 2; according to liver fibrosis stage F0-3 (top) versus cirrhosis (bottom).

Liver stiffness measured by 2D-SWE and TE increased after the moderate calorie Meal A (Fig 2) and after the high calorie Meal B (both p<0.01 for baseline versus peak liver stiffness). Liver stiffness increased at median by 22% for 2D-SWE and 28% for TE after Meal A (Table 2); and 31% for 2D-SWE and 37% for TE after Meal B. Patients with cirrhosis experienced the most pronounced increase in liver stiffness (Fig 3). When comparing the two different sized meals, there was no significant difference between the increase in liver stiffness: After Meal A, 2D-SWE increased 2.0 kPa, versus 3.9 kPa after Meal B (p = 0.096). For TE, the increase was 2.4 kPa versus 2.6 kPa (p = 0.134) (Table 2, Fig 3). After both meals, the average time to peak liver stiffness was 60 minutes (Table 2), but in ten patients, half of whom had cirrhosis, liver stiffness peaked at 180 minutes after both meals and with both elastography techniques.

**Table 2:** Changes in liver stiffness, controlled attenuation parameter and spleen stiffness after a moderate (Meal A) and high caloric (Meal B) meal.

	Meal A			Meal B		
	All (n = 60)	F0-3 (n = 28)	F4 (n = 32)	All (n = 60)	F0-3 (n = 28)	F4 (n = 32)
**Liver stiffness**						
**2D-SWE**						
Baseline value (kPa)	13.6 ±24.1	7.1 ±3	30.1 ±18.6	12.9 ±26.0	6.7 ±2.7	31.9 ±15.9
Peak value (kPa)	22.2 ±33.8	8.2 ±4.3	39.2 ±21.5	17.1 ±31.9	8.0 ±5.6	39.9 ±19.7
Delta* (kPa)	2 ±8.2	1.0 ±1.4	7.4 ±9.9	3.9 ±8.3	1.8 ±2.2	9.8 ±9.9
% difference (%)	20.1 ±27.8	13.3 ±18.9	26.0 ±34.0	30.5 ±30.7	29.9 ±34.0	38.9 ±37.8
Time to peak value (minutes)	60 ±80	60 ±80	60 ±140	60 ±80	40 ±100	60 ±80
**TE**						
Baseline value (kPa)	12.6 ±26.8	5.6 ±4.1	32.4 ±28.3	10.7 ±29.0	5.8 ±3.5	36.1 ±26.7
Peak value (kPa)	19.8 ±46.4	7.0 ±4.6	53.3 ±10.0	13.2 ±42.1	7.8 ±6.0	53.3 ±39.7
Delta* (kPa)	2.4 ±7.9	1.3 ±1.3	10.5 ±12.9	2.6 ±8.4	2.2 ±1.4	11.2 ±13.6
% difference (%)	28 ±30	23 ±23	42.3 ±37.7	37.2 ±40.4	36.9 ±32.4	39.3 ±60.3
Time to peak value (minutes)	60 ±80	60 ±100	60 ±60	60 ±100	60 ±100	120 ±120
**Spleen stiffness**						
**2D-SWE**						
Baseline value (kPa)	40.1 ±24.0	23.3 ±5.1	40.7 ±14.2	28.5 ±30.1	19.5 ±10.1	48.1 ±28.4
Peak value (kPa)	49.6 ±23.7	25.9 ±14.4	50.7 ±28.5	42.6 ±21.4	25.9 ±13.9	49.5 ±30.1
Delta* (kPa)	5.3 ±13.3	5.4 ±13.3	5.3 ±11.7	6.4 ±11.0	8.1 ±8.8	6.4 ±13.3
**TE**						
Baseline value (kPa)	46.4 ±44.0	26.3 ±9.0	61.6 ±28.6	47.2 ±43.7	24.1 ±21.1	62.7 ±27.8
Peak value (kPa)	67.8 ±29.3	42.5 ±34.5	73.5 ±9.0	70.0 ±32.8	36.8 ±15.2	75.0 ±5.0
Delta* (kPa)	10.0 ±20.6	16.8 ±24.6	7.1 ±17.7	8.9 ±15.0	10.7 ±11.6	8.6 ±15.0
2D-SWE and TE combined**						
% difference (%)	17 ±55	38 ±69	10 ±51	19 ±56	35 ±41	18 ±56
Time to peak value (minutes)	90 ±120	120 ±120	60 ±120	60 ±140	60 ±100	60 ±140
**Steatosis**						
**CAP**						
Baseline value (dB/min)	253.0 ±81.0	280.5 ±71.0	243.0 ±79.5	268.0 ±69.0	282.0 ±82.0	255.0 ±77.0
Peak value (dB/min)	270.5 ±92.0	301.5 ±94.0	251.0 ±55.5	290.0 ±83.0	320.5 ±84.0	267.0 ±98.0
Delta* (dB/min)	22.0 ±38.5	19.5 ±32.0	26.5 ±55.0	22.0 ±54.0	19.5 ±55.0	23.5 ±49.0
% difference (%)	7.4 ±15.2	7.4 ±12.6	7.1 ±18.5	9.9 ±19.1	9.1 ±20.8	10.3 ±17.1
Time to peak value (minutes)	60 ±80	60 ±80	60 ±140	120 ±120	120 ±160	180 ±120

2D-SWE, real-time 2-dimensional shear wave elastography; TE, transient elastography; CAP, controlled attenuation parameter.

*Delta denotes the difference between baseline and peak value.

**The proportional changes in spleen stiffness was combined for the two techniques to adjust for a low number of successful measurements.

We considered an increase in liver stiffness of 20% or more clinically relevant. After Meal A, 24% (2D-SWE) and 29% (TE) of patients had persistently elevated liver stiffness of 20% or more from baseline, three hours after meal ingestion. After Meal B, it was 31% (2D-SWE) and 50% (TE) of patients (tests for difference between meals, p = 0.07). The majority of patients with elevated liver stiffness beyond three hours had cirrhosis (range 45–75%, depending on elastography technique and meal size).

We confirmed the above findings in subgroup analyses including only patients with alcoholic liver disease and according to abstinence or active drinking (data not shown).

### Misclassifications of fibrosis stage and clinically significant portal hypertension by liver stiffness

The temporary increase in liver stiffness after the moderate and high caloric meal caused 22% and 34% of patients to be misclassified with 2D-SWE (p = 0.225), and 20% and 38% with TE (p = 0.021).

In the fibrosis patients, Meal A caused overestimation of fibrosis stage in 36% of patients assessed with 2D-SWE and in 24% of patients assessed with TE. The high calorie Meal B caused overestimation of fibrosis stage in a higher proportion of F0-3 patients: 52% with 2D-SWE and 54% with TE. In the cirrhosis patients, the moderate calorie Meal A caused overestimation of clinically significant portal hypertension assessed with liver stiffness in 10% (2D-SWE) and 14% (TE) of patients, versus 13% (2D-SWE) and 16% (TE) caused by the high calorie meal. Of the misclassified patients, 73% had a peak increase in 2D-SWE liver stiffness ≥20% from baseline; versus 54% of the correctly classified patients. With TE, 93% of the misclassified patients increased ≥20% from baseline (Table 3). Three hours after Meal A, four (2D-SWE) and three (TE) patients were still misclassified with a liver stiffness ≥20% of baseline. For the high calorie meal, Meal B, four (2D-SWE) and nine (TE) patients were still misclassified with a liver stiffness ≥20% of baseline after three hours. In logistic regression analysis, the high calorie meal significantly predicted misclassifications with TE, independent of fibrosis stage (Table 4).

**Table 3:** Misclassification of fibrosis amount.

	Meal A		Meal B	
	Total misclassified	Misclassified and ≥20% increase from baseline	Total misclassified	Misclassified and ≥20% increase from baseline
**All patients**				
**2D-SWE**	22%	16%	35%	26%
**TE**	20%	18%	39%	36%
**CAP**	26%	9%	33%	15%
**METAVIR F0-3**				
**2D-SWE**	36%	21%	52%	48%
**TE**	25%	21%	53%	50%
**CAP**	46%	18%	36%	27%
**METAVIR F4**				
**2D-SWE**	5%	5%	30%	20%
**TE**	14%	14%	16%	16%
**CAP**	12%	12%	29%	29%

Percentage of misclassified patients and percentage of misclassified patients with a concomitant increase ≥20% from baseline after a moderate (meal A) and high (meal B) caloric meal.

2D-SWE, real-time 2-dimensional shear wave elastography; TE, transient elastography; CAP, controlled attenuation parameter.

**Table 4:** Predictors of misclassifications and increases ≥20% from baseline in univariable analyses.

	Hazard ratio	95% confidence interval	p
**Liver stiffness with 2D-SWE**			
**Misclassification**			
Cirrhosis	0.18	0.05–0.61	0.006
High calorie meal	1.99	0.76–5.23	0.164
**Liver stiffness increase ≥20%**			
Cirrhosis	5.14	1.72–15.349	0.053
High calorie meal	2.54	0.94–6.87	0.065
Liver stiffness with TE			
**Misclassifications**			
Cirrhosis	0.20	0.04–1.12	0.068
High calorie meal	5.03	1.14–22.22	0.033
**Liver stiffness increase ≥20%**			
Cirrhosis	1.19	0.29–4.91	0.814
High calorie meal	1.87	0.64–5.42	0.251
**Controlled attenuation parameter**			
**Misclassifications**			
Cirrhosis	0.31	0.09–1.11	0.072
High calorie meal	1.35	0.48–3.77	0.564
**CAP increase ≥20%**			
Cirrhosis	1.19	0.33–4.26	0.793
High calorie meal	1.01	0.33–3.14	0.983

Univariate logistic regression controlling for repeated investigations in the same patients on two consecutive days. 2D-SWE, real-time 2-dimensional shear wave elastography; TE, transient elastography; CAP, controlled attenuation parameter.

### The effect of a meal on spleen stiffness

Because of low success rates for spleen stiffness with 2D-SWE and TE (24% and 60% successful baseline measurements, respectively) we merged data on the proportional increase in spleen stiffness for the two techniques. This allowed us to analyze post-meal, proportional changes in spleen stiffness in 36 patients after Meal A and 32 patients after Meal B. Overall, spleen stiffness increased significantly from baseline to peak post-meal value (Table 2; p<0.001) on both study days. The proportional increase from baseline was 17% after Meal A and 19% after Meal B (difference between days p = 0.245). In contrast to liver stiffness, spleen stiffness increased proportionally more in the F0-3 fibrosis group than in the cirrhosis group (38% for F0-3 fibrosis vs. 10% for cirrhosis, p = 0.084 for Meal A; 35% vs. 19% p = 0.883 for Meal B).

### Correlation with portal blood velocity and portal blood flow

**Fig 4:**
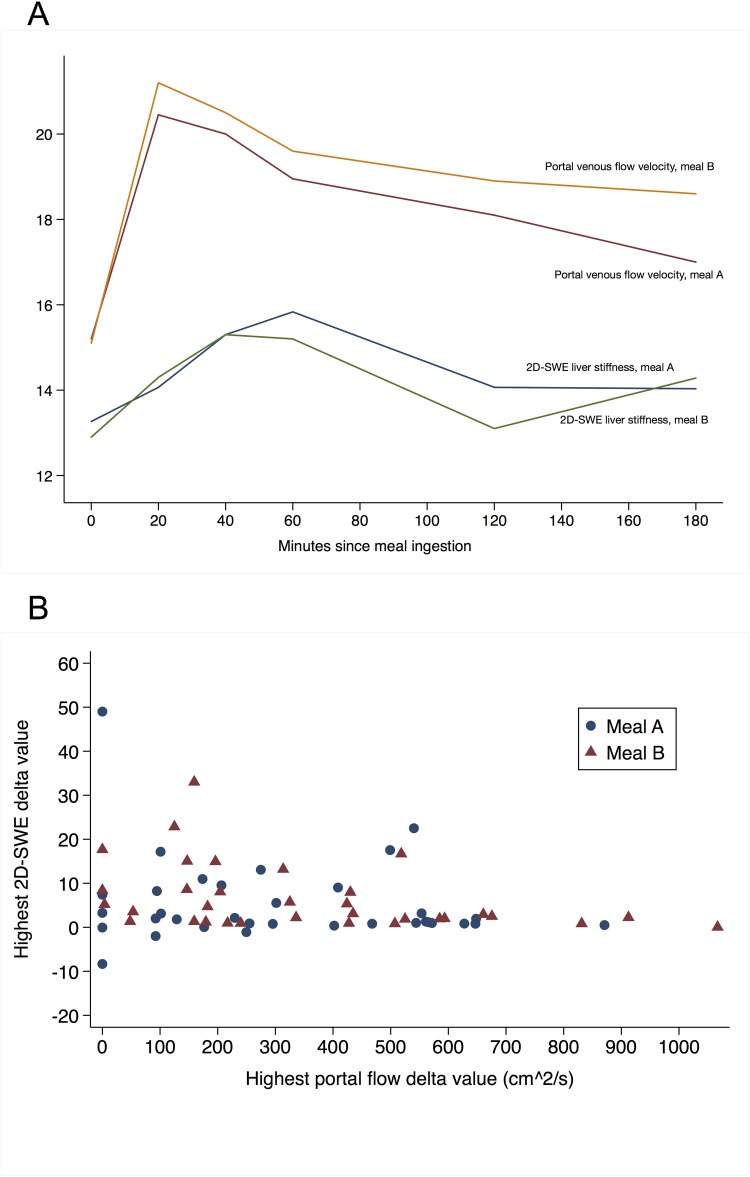
Liver stiffness and portal venous flow velocity. A) Meal-induced changes in liver stiffness (kPa) and portal venous flow velocity (cm/s). B) Correlation between liver stiffness (kPa) and portal venous flow velocity (cm/s).

Portal venous blood velocity increased 35% and flow increased 48% from baseline within an average of 40 minutes after meal ingestion (p<0.001). There was no significant difference in the increase in velocity or flow between a high and a low calorie meal. Both velocity and flow increased significantly more in patients with F0-3 fibrosis, than in cirrhosis patients. While the increase in portal blood velocity preceded the rise in liver stiffness (Fig 4), increased liver stiffness did not correlate significantly with increased portal velocity or flow, neither in univariate analysis, or when adjusting for degree of fibrosis.

### Controlled attenuation parameter is influenced by meal ingestion

Controlled Attenuation Parameter (CAP) increased slightly after a moderate calorie and a high calorie meal (Table 2). The overall increase in CAP was 22 dB/m after Meal A and 22 dB/m after Meal B (p = 0.603 between study days), with a proportional increase of 7.4% and 9.9% after Meal A and Meal B, respectively. The degree of fibrosis did not influence baseline or peak CAP (all p>0.6). The highest CAP was observed after 60 minutes after Meal A and 120 minutes after Meal B.

Controlled attenuation parameter increased above the 250 dB/m and 300 dB/m thresholds, causing steatosis misclassifications, in 26% and 33% of patients after Meal A and Meal B, respectively. As opposed to liver stiffness, CAP only increased more than 20% from baseline in 17% of patients, across study days.

## Discussion

In this study food intake caused up to half of the non-cirrhotic patients to be misclassified with higher stages of fibrosis and 10–14% of cirrhotic patients to be misclassified with clinically significant portal hypertension. Liver stiffness, spleen stiffness and controlled attenuation parameter all increased after a moderate and high calorie meal, assessed with both transient and 2-dimensional shear wave elastography. Liver stiffness remained substantially elevated from baseline in 24–50% of patients three hours after the meal, depending on elastography technique and meal size. We also observed that one in six patients did not peak before 180 minutes, equally distributed among F0-3 fibrosis patients and cirrhosis patients.

Our finding supports an optimal fasting time of longer than three hours. This contrasts with recent elastography guidelines[2] and with a study in chronic hepatitis C patients, where a 600 kcal meal caused a temporary increase in liver stiffness that normalized within 2 hours.[12] By utilizing a longer period of observation after the meal, we demonstrate that several patients has more than 20% elevated liver stiffness 3 hours after the meal, causing false positive results and misclassifications. This finding was observed for both the moderate and high calorie meal, across fibrosis groups. We are thereby in line with a study in chronic hepatitis B patients, where an 850 kcal meal caused 18% unreliable liver stiffness measurements 120 minutes after the meal.[25]

A meal of higher caloric content and volumetric size increased the proportion of misclassifications, but only significantly for patients assessed with TE. Overall, the meal-related increases in liver stiffness was only moderately affected by the size of the meal; while spleen stiffness, CAP and time to peak was unaffected by the meal size. This may relate to the fact that some of the factors driving changes in hepatic viscoelasticity after a meal are independent of meal size. For example, portal venous velocity and flow were not predictors of liver stiffness increase; despite both variables being elevated after a meal. This is in agreement with a previous study where liver stiffness increases correlated with arterial flow, but not portal flow in patients with cirrhosis.[13] However, another study suggested a linear relationship between liver stiffness and portal flow in non-cirrhotic patients.[26]

The effect of a meal on liver stiffness is similar across liver disease etiology and ultrasound elastography technique. Prior studies have assessed only patients with chronic viral hepatitis, used only TE for assessment of liver stiffness and only evaluated patients for one or two hours after the meal. In this study, we are the first to include a population primarily consisting of alcoholic patients, the first to investigate liver and spleen stiffness with both TE and 2D-SWE and the first to investigate meal-related changes in CAP.

Spleen stiffness is a potential marker for clinically significant portal hypertension and the presence of esophageal varices. It is therefore of interest, that spleen stiffness also seem to increase after a meal. However, the high number of invalid measurements, possibly due to normal spleen size in the majority of patients, questions the generalizability of our results and the clinical applicability of spleen stiffness assessment. In this study, in contrast to liver stiffness, the proportional spleen stiffness increase was comparable between fibrosis groups. A possible explanation was that eight patients with cirrhosis had a spleen stiffness above 70 kPa measured by TE, which prohibits a large proportional increase due to an upper TE limit of 75 kPa.

Our study also suggests that meal intake affects the attenuation of liver tissue, thereby causing postprandial increases in CAP values. While the average peak post meal value was only 36 dB/m above baseline, it was enough to elevate CAP across common thresholds for steatosis classification in 26% of patients after Meal A and 33% of patients after Meal B.

In conclusion, we recommend more than three-hours fasting before the assessment of liver stiffness, spleen stiffness and controlled attenuation parameter. Our data suggest that this approach should be exercised across the entire spectrum of fibrosis, across different ultrasound elastography techniques and for patients of all liver disease etiologies.
